# An Investigation of Non-Coplanar Volumetric Modulated Radiation Therapy for Locally Advanced Unresectable Pancreatic Cancer Using a Trajectory Optimization Method

**DOI:** 10.3389/fonc.2021.717634

**Published:** 2021-09-17

**Authors:** Gong Wang, Hao Wang, Hongqing Zhuang, Ruijie Yang

**Affiliations:** Department of Radiation Oncology, Peking University Third Hospital, Beijing, China

**Keywords:** pancreatic cancer, non-coplanar, VMAT, trajectory optimization, treatment planning

## Abstract

**Purpose:**

This study was conducted in order to develop a trajectory optimization algorithm for non-coplanar volumetric modulated arc therapy (VMAT) and investigate the potential of organs at risk (OARs) sparing in locally advanced pancreatic cancer patients using non-coplanar VMAT.

**Methods and Materials:**

Firstly, a cost map that represents the ray–OAR voxel intersections at each source position was generated using a ray-tracing algorithm. A graph search algorithm was then used to determine the least-cost path from the cost map. Lastly, full arcs or partial arcs were selected based on the least-cost path to generate the non-coplanar VMAT (ncVMAT) trajectories. Clinical coplanar VMAT (coVMAT) plans for 11 patients diagnosed with locally advanced unresectable pancreatic cancer (LAPC) receiving 45 to 70 Gy in 25 fractions were replanned using non-coplanar VMAT trajectories. Both coplanar and non-coplanar plans were normalized to cover 95% of the PTV_45 Gy_ volume with a prescription dose of 45 Gy. The conformity index (CI), homogeneity index (HI), PTV coverage, and dose to the OARs were compared between coVMAT and ncVMAT plans.

**Results:**

With ncVMAT, the mean coverage of PTV_50 Gy_, PTV_54 Gy_, PTV_60 Gy_, and PTV_70 Gy_ increased significantly. The mean conformity index of PTV_45 Gy_, PTV_54 Gy_, and PTV_70 Gy_ was also improved in the ncVMAT plans. Compared with coVMAT plans, the ncVMAT plans resulted in significantly lower doses to the spinal cord, bilateral kidneys, stomach, and duodenum. The maximum dose to the spinal cord decreased by 6.11%. The mean dose to the left and right kidneys decreased by an average of 5.52% and 11.71%, respectively. The *D*_max_, *D*_mean_, and *D*_15%_ of the stomach were reduced by an average of 7.45%, 15.82%, and 16.79%, separately. The *D*_15%_ and *D*_mean_ of the duodenum decreased 6.38% and 5.64%, respectively.

**Conclusion:**

A trajectory optimization algorithm was developed for non-coplanar VMAT. Compared with conventional coplanar VMAT, non-coplanar VMAT resulted in improved coverage and conformity to the targets. The sparing of OARs was significantly improved in non-coplanar VMAT compared with coVMAT plans for locally advanced pancreatic cancer.

## Introduction

According to the American Cancer Society, pancreatic cancer was the seventh leading cause of cancer-related death worldwide for both genders in 2018 (1). The only curative therapy for patients diagnosed with pancreatic cancer is surgical resection. Nevertheless, only 15%–20% of these patients presented with resectable tumors (2). The standard care for patients with unresectable pancreatic cancer is chemotherapy and/or chemoradiotherapy (3). However, conventionally fractionated radiotherapy which delivers 40 to 60 Gy in 1.8–2.0 Gy per fraction showed minimal to no local tumor control benefit for patients with locally advanced pancreatic cancer (LAPC) (4). With the development of image-guided radiotherapy and immobilization techniques, stereotactic body radiotherapy (SBRT) which precisely delivers a high dose per fraction has become a promising option for the treatment of patient with LAPC. A study that investigated the outcomes of patients treated with SBRT using the National Cancer Data Base (NCDB) showed that the median overall survival (OS) (13.9 *vs.* 11.6 months) and the 2-year OS rate (21.7% *vs.* 16.5%) were significantly higher with SBRT *versus* conventionally fractionated radiation therapy (5). The reason why SBRT resulted in a better survival rate is that SBRT allows to deliver a higher biological effective dose (BED) to patients and higher BED is associated with better local control. On the other hand, early studies implementing SBRT were associated with high early and/or late gastrointestinal side effects (6, 7). Therefore, the sparing of radiosensitive gastrointestinal organs such as the stomach, small intestines, colon, and duodenum near the pancreas becomes the main difficulty of dose escalation in LAPC patients (4).

A lot of studies have been done to investigate the application of non-coplanar radiotherapy in OAR dose sparing for different sites. Evidence has shown that non-coplanar plans were superior in normal tissue sparing compared with the conventional coplanar technique (8–12). Smyth et al. evaluated non-coplanar VMAT for OAR sparing in primary brain tumor radiotherapy (13). They found that compared with coplanar VMAT, non-coplanar VMAT significantly reduced doses to the contralateral lobe, optic nerve, hippocampus, and temporal lobe for patients with primary brain tumors. Yu et al. reported the first clinical implementation of 4π radiation therapy in recurrent high-grade glioma patients (14). Twenty beam orientations were used for optimization. The results showed that compared with coplanar VMAT, 4π IMRT plans resulted in equal or significantly lower OAR doses. A study shows that in head and neck cases, non-coplanar IMRT can significantly reduce dose to hippocampi and whole brain which helps preserve neurocognitive function (15). Another study of non-coplanar VMAT for brain metastases shows that non-coplanar VMAT generated more rapid dose falloff and higher conformity compared with co-planar VMAT (8). Uto et al. compared coplanar VMAT and non-coplanar VMAT for hippocampus protection in craniopharyngioms cases and found that non-coplanar VMAT significantly reduced the dose to bilateral hippocampus (16). The two non-coplanar arcs were set at couch angles of 45° and 315°. Similarly, Cheung et al. applied non-coplanar VMAT to primary brain tumor using non-coplanr arcs with couch rotated to 45° or 315° (17). An alternative approach to non-coplanar IMRT or non-coplanar VMAT is the VMAT+ technique proposed by Sharfo et al. which combined full coplanar arcs with few non-coplanar beams with optimized beam angles (18). The advantage of the VMAT+ technique is that it has similar plan quality to fully non-coplanar IMRT but much shorter treatment delivery time.

Non-coplanar techniques have also been implemented in the management of pancreatic cancer. Chang et al. found that non-coplanar IMRT was able to significantly decrease bilateral kidney dose for unresectable pancreatic cancer compared with coplanar IMRT (19). The non-coplanar IMRT beam angles were manually selected in beam’s eye view (BEV) to spare the kidneys. Osborne et al. also found that the non-coplanar plans show overall benefits over coplanar plans (20). Two lateral wedged oblique fields and two wedged non-coplanar oblique fields were used. Burghelea et al. investigated non-coplanar trajectories for LAPC cases using dynamic wave arc on the VERO gimballed linac (21). However, the beam directions were selected by a human planner. Previous studies on pancreatic cancer were based on the IMRT technique or the beam angles were selected manually due to the complexity of beam orientation optimization (22). In this study, we present an implementation of non-coplanar VMAT technique which utilized cost map generation and trajectory optimization algorithm for OAR dose sparing in LAPC patients.

## Materials and Methods

### Patient Selection

This retrospective study was approved by the institutional review board and informed consent was waived. Eleven patients with locally advanced pancreatic cancer treated with conventional coplanar VMAT plans were selected in our study. Patients were simulated using Philips Brilliance Big-Bore helical CT with 3 mm slice thickness. Plans were designed and optimized using VMAT techniques with simultaneous integrated boost (SIB) in the Eclipse treatment planning system (Varian Medical System, Palo Alto, CA, USA). PTV prescription dose includes 45 Gy in 1.8 Gy fractions, 50 Gy in 2 Gy fractions, 54 Gy in 2.14 Gy fractions, 60 Gy in 2.4 Gy fractions, and 70 Gy in 2.8 Gy fractions. The average planning target volume (PTV) size was 435.31 cm^3^ for PTV_45 Gy_, 215.32 cm^3^ for PTV_50 Gy_, 45.50 cm^3^ for PTV_54 Gy_, 43.53 cm^3^ for PTV_60 Gy_, and 20.37 cm^3^ for PTV_70 Gy_.

### Data Input

For each patient, the Digital Imaging and Communications in Medicine (DICOM) structure set which contains isocenter position, contours of PTV, and OARs were exported from Eclipse treatment planning system into MATLAB (The MathWorks, Natick, MA, USA) to generate a 3D patient phantom for ray-tracing simulation.

### Ray Tracing and Cost Map Generation

The gantry rotated from 0° to 360° with control points spaced every 2°, and the couch rotated from 270° to 90° with control points spaced every 2°. For each source position determined by the couch and gantry angle, ray tracing was performed using a MATLAB algorithm (23). The couch and gantry angle combinations which may cause collision were eliminated from ray tracing. The collision regions were determined by measuring the permitted gantry rotation angle for each couch angle with a patient phantom placed on the couch.

For each source position, ray tracing was performed, and a list of coordinates in three dimensions of the intersected voxels was produced. Then, the number of intersected voxels in different types of OARs was calculated. The OARs evaluated in this study were the small intestines, duodenum, colon, liver, bilateral kidneys, stomach, and spinal cord.

**Figure 1:**
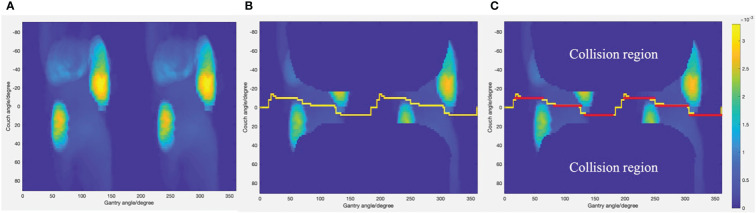
**(A)** Cost map generated through ray-tracing simulation. **(B)** The least cost trajectory generated by Dijkstra’s algorithm displayed on cost map with collision region excluded. **(C)** The subarcs with fixed couch positions (displayed as red lines) adopted as final non-coplanar trajectories.

For each source position determined by couch angle *c* and gantry angle *g*, the cost of each node *C_c,g_* is given by the sum of the relative volume of each OAR intersected during ray tracing:


(1) $$C_{c,g}=\Sigma_{v\in V}\frac{n_{v}}{N_{v}}$$

where *v* is the OAR of interest, *V* is the set of all OARs evaluated, *n_v_* is the number of organ *v* voxels intersected by the ray, and *N_v_* is the total number of voxels of organ *v*. After the cost of each permitted source position was calculated, the cost data were reformatted into a matrix and displayed as a cost map as shown in **Figure 1A**.

### Trajectory Selection

After the cost map was generated, the trajectory optimization was simplified as a traveling salesman problem. A salesman wants to travel between several cities to sell goods. The distance to each city is different and not every city is connected. The problem is to find the shortest route between a certain number of destinations that must be visited. One constraint of the traveling salesman problem is that each city can be visited only once. Similar to the salesman problem, we want to find the least-cost trajectory through various nodes. The cost of each node is similar to the distance to each city in the traveling salesman problem. Dijkstra’s algorithm was used to find the minimal cost trajectory through the cost map. Dijkstra’s algorithm aims to find the shortest paths from a starting node to all other nodes in a weighted graph. This algorithm was created by the Dutch computer scientist Dr. Edsger W. Dijkstra. In this study, we only need to find the shortest path from the source node to a target node. The source node was at gantry angle of 0 and couch angle of 0. The target node was at gantry angle of 360 and couch angle of 0. The reason of doing this is that every gantry angle from 0° to 360° will be visited. There are four inputs when implementing Dijkstra’s algorithm. The first input is the cost of moving from node *i* to node *j* which is the sum of the cost of node *i* and node *j*. The second input is an adjacency matrix which indicates which nodes are adjacent to which other nodes in the cost map:


(2) $$A(i,j)=\{\begin{array}{c} 1,\;if\;g_{j}-g_{i}=2{}^{\circ}\;or\;\left|c_{i}-c_{j}\right|=2{}^{\circ}\\ 0,else \end{array}$$

where *g_i_* and *g_j_* are the gantry angle of node *i* and node *j*, and *c_i_* and *c_j_* are the couch angel of node *i* and node *j*. The last two inputs are the coordinates of the source node and target node. The outputs are the minimal cost path connecting the starting node and ending node and the cost value for the path. **Figure 1B** shows the least-cost path generated by Dijkstra’s algorithm. Due to restrictions of the Eclipse treatment planning system, the couch and gantry cannot rotate simultaneously for VMAT plans. Therefore, the optimized trajectory was divided into several subarcs with fixed couch positions as shown in **Figure 1C**. Subarcs with an arc length less than 30° cannot be optimized in the treatment planning system and, therefore, were excluded from plan optimization.

### Treatment Planning

The original clinical coVMAT plans consisted of two coplanar arcs with control points spaced every 2°. Plans were designed and optimized using the Eclipse planning system. Both the coplanar and non-coplanar plans were normalized to cover 95% of PTV_45 Gy_ volume with a prescription dose of 45 Gy. Coplanar and non-coplanar plans were produced for a 6MV Varian Trilogy linear accelerator with a Millennium 120 leaf MLC. The minimal MLC width was 5 mm. The dose calculation algorithm was the anisotropic analytical algorithm (AAA). The calculation resolution was 2.5 mm.

### Plan Evaluation

Multiple dose metrics were evaluated for coVMAT and ncVMAT plans. For the targets, homogeneity index (HI), conformity index (CI), and mean coverage (V100%) were compared.

The HI was defined as:


(3) $$\text{HI}=\frac{D_{5\%}}{D_{95\%}}\times 100,$$

where *D*_5%_ and *D*_95%_ are the doses to 5% and 95% of the PTV volume.

CI was recommended by Paddicks’s formula:


(4) $$\text{CI}=\frac{V(PI\cap T)}{V(T)}\times \frac{V(PI\cap T)}{V(PI)},$$

where *V*(*PI* ∩ *T*) is the volume of the intersection of the prescription isodoses and the target, *V*(*T*) is the volume of the target, and *V*(*PI*) is the volume of the prescription isodose. For OARs, the maximum point dose (*D*_max_), dose to 15% of OAR volume (*D*_15%_), dose to 2 cm^3^ of OAR volume (*D*_2 cc_), the portion of volume receiving 20 Gy (*V*_20 Gy_), and 40 Gy (*V*_40 Gy_) were evaluated for the small intestine, colon, and stomach. The mean dose (*D*_mean_) was evaluated for bilateral kidneys. *D*_mean_ was evaluated for the liver. The *D*_max_ was also evaluated for the spinal cord. *D*_max_ and *D*_15%_ were evaluated for the duodenum. Those criteria were selected according to RTOG 0848 and practice in our institution (24). **Table 1** shows the normal tissue dose constraints for conventionally fractionated radiotherapy from RTOG 0848. Monitor unit (MU) was calculated for each plan. Paired *t*-test was used to perform statistical analysis, and a significance level of *P≤*0.05 (two-tailed) was used.

**Table 1:** Normal tissue dose constraints.

Structure	RTOG 0848
Spinal cord	Max dose <4,500 cGy
Liver	Mean dose ≤2,500 cGy
Small bowel	Max dose <5,400 cGy
	*D*_15%_ ≤4,500 cGy
Stomach	Max dose <5,400 cGy
	*D*_15%_ ≤4,500 cGy
Bilateral kidneys	Mean dose ≤1,800 cGy

## Results

The dosimetric parameters of the PTVs are shown in **Table 2**. Both coVMAT and ncVMAT plans were normalized to over 95% of the PTV_45 Gy_ volume with the prescription dose of 45 Gy. Therefore, the mean coverage of PTV_45 Gy_ was both 95% for coVMAT and ncVMAT plans. Compared with coVMAT plans, the mean coverage of PTV_50 Gy_, PTV_54 Gy_, PTV_60 Gy_, and PTV_70 Gy_ of ncVMAT plans were significantly increased. The mean conformity index of PTV_45 Gy_, PTV_54 Gy_, and PTV_70 Gy_ was also increased, while the mean conformity index of PTV_50 Gy_ and PTV_60 Gy_ decreased slightly. There was no significant difference for homogeneity indexes between the coVMAT and ncVMAT plans.

**Table 2:** Dosimetric parameters of targets for coVMAT and ncVMAT plans.

	Coplanar	Non-coplanar
PTV_45 Gy_ CI	0.87 ± 0.07	0.91 ± 0.12
PTV_45 Gy_ HI	1.41 ± 0.14	1.43 ± 0.15
PTV_45 Gy_ V100%	95%	95%
PTV_50 Gy_ CI	0.77 ± 0.10	0.68 ± 0.15
PTV_50 Gy_ HI	1.37 ± 0.14	1.38 ± 0.15
PTV_50 Gy_ V100%	89.08% ± 6.93%	93.07% ± 3.98%
PTV_54 Gy_ CI	0.34 ± 0.25	0.42 ± 0.22
PTV_54 Gy_ HI	1.02	1.02
PTV_54 Gy_ V100%	44.35% ± 34.35%	62.95% ± 36.45%
PTV_60 Gy_ CI	0.84 ± 0.17	0.73 ± 0.14
PTV_60 Gy_ HI	1.14 ± 0.08	1.14 ± 0.08
PTV_60 Gy_ V100%	96% ± 4.34%	97% ± 2.86%
PTV_70 Gy_ CI	0.73 ± 0.12	0.84 ± 0.24
PTV_70 Gy_ HI	1.04 ± 0.01	1.05 ± 0.01
PTV_70 Gy_ V100%	89.25% ± 10.68%	97.38% ± 2.54%

The dosimetric parameters of the OARs are shown in **Table 3**. Compared with coVMAT plans, the ncVMAT plans resulted in a lower dose to the liver. The mean dose to the liver was reduced by an average of 8.8%. Compared with coVMAT plans, ncVMAT plans resulted in lower doses to the spinal cord. The max dose to the spinal cord was reduced by an average of 6.11%. The ncVMAT plans also resulted in lower doses to bilateral kidneys. The mean dose to the left kidney decreased by an average of 5.52%. The mean dose to the right kidney decreased by an average of 11.71%. For the stomach, a significant improvement of the *D*_max_, *D*_mean_, and *D*_15%_ was observed. For the small intestines, ncVMAT resulted in lower *D*_max_, *D*_mean_, *D*_2 cc_, and *V*_20 Gy_, while *V*_40 Gy_ increased slightly. There was no significant difference between coVAMT and ncVAMT for small intestine *D*_15%_. The *D*_mean_ and *V*_20 Gy_ of the colon decreased, while the colon *V*_40 Gy_ increased. Colon *D*_max_, *D*_2 cc_, and *D*_15%_ were similar for coVMAT and ncVMAT. Compared with coVMAT plans, ncVMAT plans also resulted in lower *D*_15%_ and *D*_mean_ to the duodenum.

**Table 3:** Normal tissue dose–volume statistics.

Dose metrics	Coplanar	Non-coplanar	*P*
Liver mean dose (Gy)	11.55 ± 12.51	10.66 ± 12.27	0.07
Rt kidney mean dose (Gy)	9.14 ± 3.96	8.16 ± 3.73	0.020
Lt kidney mean dose (Gy)	12.53 ± 3.37	11.74 ± 2.99	0.035
Spinal cord max dose (Gy)	37.59 ± 6.99	35.02 ± 5.95	0.067
Small intestine max dose (Gy)	51.59 ± 3.39	50.21 ± 3.58	0.029
Small intestine mean dose (Gy)	12.96 ± 7.72	12.08 ± 7.07	0.051
Small intestine *D*_2 cc_ (Gy)	45.50 ± 7.74	43.96 ± 8.82	0.035
Small intestine volume >20 Gy (cm^3^)	136.14 ± 100.35	123.06 ± 86.45	0.110
Small intestine volume >40 Gy (cm^3^)	31.72 ± 36.92	32.95 ± 35.80	0.495
Small intestine *D*_15%_ (Gy)	24.44 ± 13.16	24.52 ± 13.35	0.878
Colon max dose (Gy)	46.56 ± 8.74	46.09 ± 9.47	0.327
Colon mean dose (Gy)	12.93 ± 4.19	11.89 ± 3.85	0.160
Colon *D*_2 cc_ (Gy)	40.74 ± 8.79	40.82 ± 8.89	0.875
Colon volume >20 Gy (cm^3^)	134.22 ± 80.70	123.99 ± 84.93	0.499
Colon volume >40 Gy (cm^3^)	7.04 ± 6.60	11.48 ± 12.04	0.108
Colon *D*_15%_ (Gy)	23.08 ± 5.98	22.96 ± 6.55	0.921
Stomach max dose (Gy)	48.34 ± 6.45	45.40 ± 9.53	0.036
Stomach mean dose (Gy)	14.40 ± 8.55	12.72 ± 8.48	0.004
Stomach *D*_15%_ (Gy)	28.01 ± 10.42	24.86 ± 12.11	0.003
Duodenum max dose (Gy)	45.40 ± 11.71	45.55 ± 11.91	0.690
Duodenum *D*_15%_ (Gy)	39.31 ± 10.83	37.65 ± 12.20	0.049
Duodenum mean dose (Gy)	30.29 ± 13.75	29.18 ± 14.37	0.062

**Figure 2:**
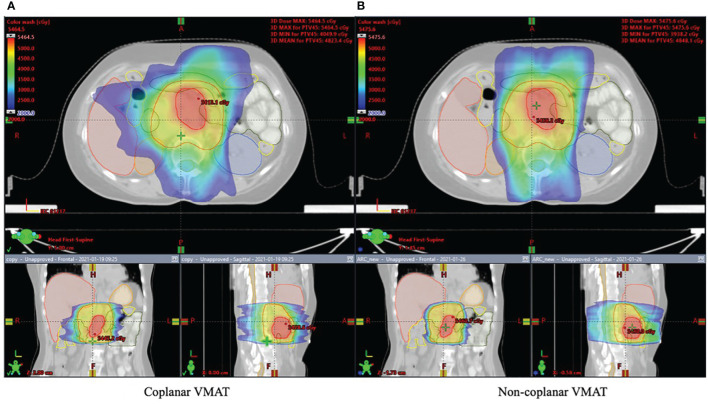
Dose distribution for one typical patient shown in axial, coronal, and sagittal views: **(A)** coplanar VMAT and **(B)** non-coplanar VMAT plan.

**Figure 3:**
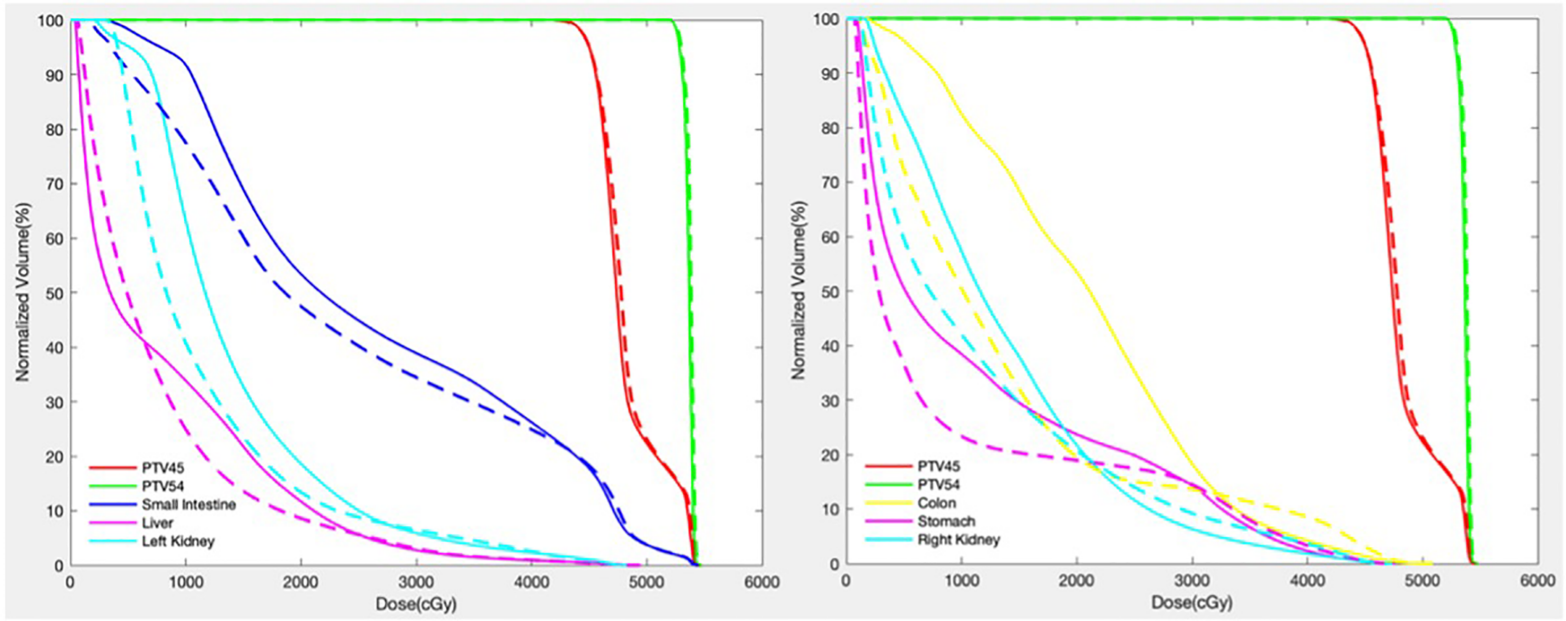
Dose–volume histogram of PTV and selected OARs (solid line—coVMAT, dashed line—ncVMAT).

**Figures 2**, **3** show the dose distribution comparison of coVMAT and ncVMAT for one typical patient and the dose–volume histogram of PTV and selected OARs. As **Figure 2** shows, the pancreas is centrally located and surrounded by many radiation-sensitive organs such as the stomach, bilateral kidneys, duodenum, colon, and small intestine. From the dose distribution, we can see that the ncVMAT plan has a better sparing of the liver, small intestine, and colon due to the additional freedom of couch rotation. The dose–volume histogram comparison also shows that ncVMAT resulted in a significant dose reduction to selected OARs with comparable target coverage to the coVMAT plan. In such a case, the improved OAR sparing can be utilized to increase the amount of dose that can be delivered to the tumor limited by normal tissue toxicity, thus improving the local control.

The average MUs for coVMAT and ncVMAT plans were 585 ± 117 and 540 ± 80 MU. The average number of subarcs for each non-coplanar plan is 10. The minimum and maximum numbers of subarcs are 8 and 12.

## Discussion

In the radiotherapy of pancreatic cancer, a hypofractionated regimen that delivers much higher BED to the tumor shows better local control compared with the conventionally fractionated regimen. However, the implementation of hypofractionated radiotherapy in pancreatic cancer is usually associated with excess normal tissue toxicity which limited the amount of radiation dose that can be delivered to the tumor. Thus, OAR sparing is of great significance in order to improve tumor control while reducing normal tissue toxicity. In this study, non-coplanar VMAT was investigated for its ability of OAR dose sparing in locally advanced pancreatic cancer patients by using a trajectory optimization approach. Due to the complexity of non-coplanar VMAT trajectory optimization, beam oreientations were selected mannually in previous studies. The quality of non-coplanar plan using manually selected beam direction largely relies on the experience of the treatment planner. This study investigates the organ at risk sparing in locally advanced pancreatic cancer patients using the non-coplanar VMAT technique. Compared with previous studies which manually select the beam orientation, the non-coplanar trajectories in our study were selected automatically using the trajectory optimization algorithm. Compared with automatic optimization methods such as the VMAT+ technique, our method is simple and easy to implement because the fluence map optimization is decoupled from beam orientation optimization. Firstly, a cost map which represents the ray–OAR overlap was produced through ray-tracing simulation for each patient. Then, a shortest pathfinding algorithm was utilized to find the minimal cost trajectory through the cost map. The result in non-coplanar trajectory was a continuous path. Due to restriction of the planning and delivery system, the linac gantry and couch cannot rotate simultaneously, and the non-coplanar VMAT trajectory was divided into several subarcs with static couch rotation.

For OARs, both the coVMAT and ncVMAT plans met the dose constraints suggested by RTOG 0848. However, ncVMAT significantly decreased the dose to the stomach, bilateral kidneys, duodenum, and spinal cord, compared with coVMAT. The stomach *D*_max_, *D*_mean_, and *D*_15%_; the bilateral kidneys *D*_mean_; the duodenum *D*_mean_ and *D*_15%_; and the spinal cord *D*_max_ were all decreased with ncVMAT. An overall lower mean dose to the small intestine and colon was also achieved with ncVMAT.

**Figure 4:**
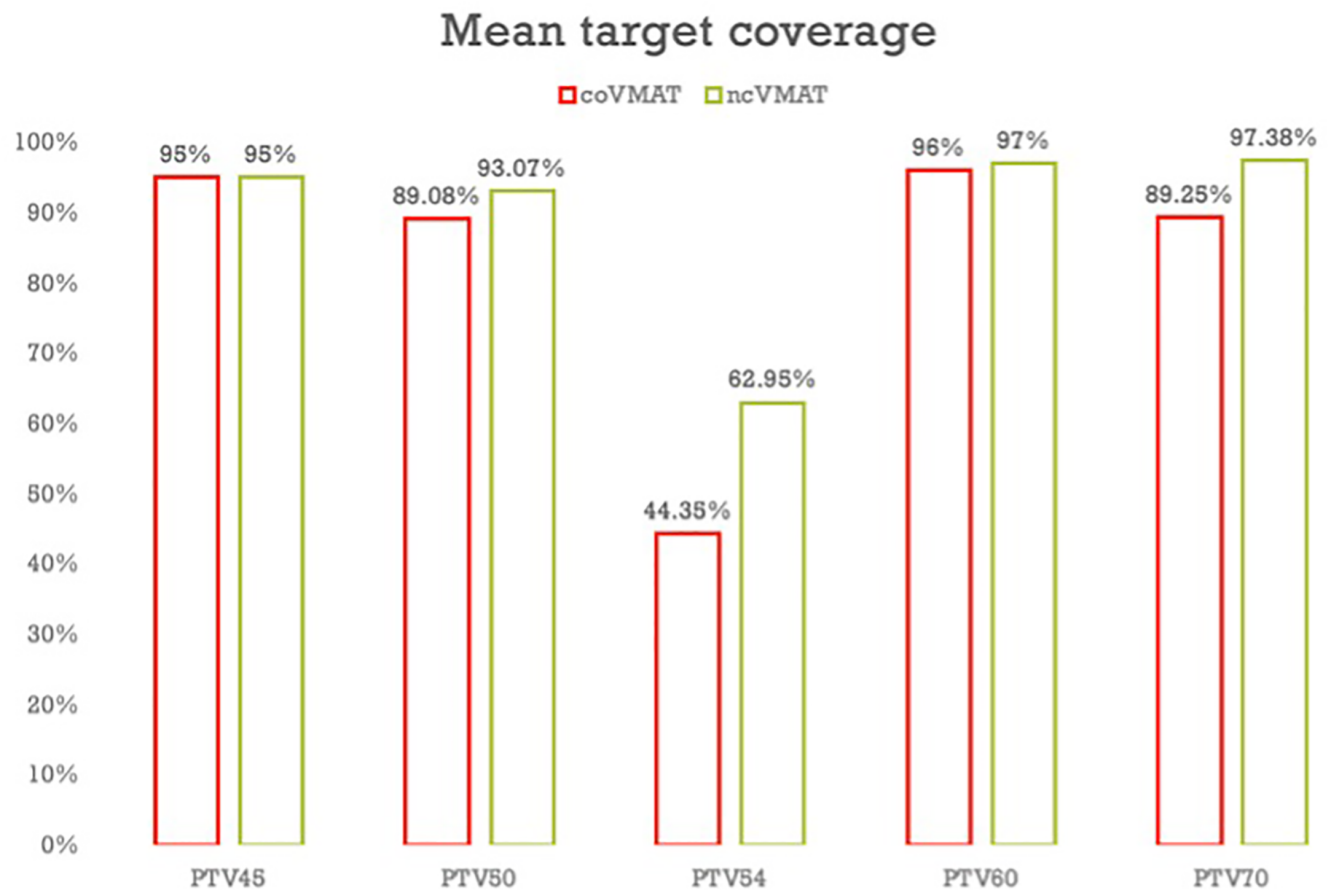
Mean target coverage comparison of coVMAT and ncVMAT plans.

As **Figure 4** shows, the non-coplanar VMAT significantly improved the mean coverage of PTV_50 Gy_, PTV_54 Gy_, PTV_60 Gy_, and PTV_70 Gy_. This could potentially improve tumor control. Improved tumor control also means improved survival. The mean conformity index of PTV_45 Gy_, PTV_54 Gy_, and PTV_70 Gy_ was also improved which indicated that ncVMAT may have a better sparing of normal tissues.

Although the MU for ncVMAT plans was slightly lower than the coVMAT plans, the treatment delivery time of ncVMAT plans can be longer than coVMAT plans due to the extra couch rotation between multiple arcs. However, the treatment time of ncVMAT plans can be reduced by implementing automatic couch rotation.

There are several limitations of this study: 1) OARs were equally weighted when generating the cost map. A customized weighting factor can be applied to each OAR according to their clinically relevant importance to generate better sparing of a specific organ. 2) Trajectory optimization was separated from VMAT plan optimization. Due to the computation complexity, the trajectory optimization was separated from VMAT plan optimization. 3) Beams that had opposite direction were equally weighted during ray tracing. The OAR can be between the source and the PTV or behind the PTV for opposite beam directions. It is less desirable to have the OAR between the source and the PTV. In such a case, the beam orientations which have OAR between the source and the PTV should be heavily penalized. 4) Treatment time and patient comfort were sacrificed by resetting the couch to multiple arc positions.

In summary, compared with conventional coplanar VMAT, non-coplanar VMAT resulted in improved coverage for PTV_50 Gy_, PTV_54 Gy_, PTV_60 Gy_, and PTV_70 Gy_. The conformity index of PTV_45 Gy_, PTV_54 Gy_, and PTV_70 Gy_ was also improved. For OARs, non-coplanar VMAT produced better dose sparing for the stomach, bilateral kidneys, duodenum, and spinal cord. A lower mean dose to the small intestine and colon was also achieved with non-coplanar VMAT with no significant difference for other OARs. In conclusion, non-coplanar VMAT has the potential of increasing the dose delivered to the tumor while reducing normal tissue toxicity, thus improving the local control of locally advanced pancreatic cancer.

## Conclusions

A trajectory optimization algorithm was developed for non-coplanar VMAT. Compared with conventional coplanar VMAT, non-coplanar VMAT resulted in improved coverage and conformity to the targets. The sparing of OARs was significantly improved in non-coplanar VMAT compared with coVMAT plans for locally advanced pancreatic cancer.

## Data Availability Statement

The original contributions presented in the study are included in the article/supplementary material. Further inquiries can be directed to the corresponding author.

## Author Contributions

All the authors have participated sufficiently in the work to take public responsibility for the appropriateness of the material and method and the collection, analysis, and interpretation of the data. All authors contributed to the article and approved the submitted version.

## Funding

This work was partly supported by the Nationl Key Research and Development Program (2021YFE0202500), Beijing Municipal Commission of Science and Technology Collaborative Innovation Project (Z201100005620012), Capital’s Funds for Health Improvement and Research (2020-2Z-40919), and China International Medical Foundation (HDRS2020030206).

## Conflict of Interest

The authors declare that the research was conducted in the absence of any commercial or financial relationships that could be construed as a potential conflict of interest.

## Publisher’s Note

All claims expressed in this article are solely those of the authors and do not necessarily represent those of their affiliated organizations, or those of the publisher, the editors and the reviewers. Any product that may be evaluated in this article, or claim that may be made by its manufacturer, is not guaranteed or endorsed by the publisher.
